# Highly active Ru/TiO_2_ nanostructures for total catalytic oxidation of propane

**DOI:** 10.1007/s11356-023-29153-w

**Published:** 2023-08-21

**Authors:** Roberto Camposeco, Omar Miguel, Ana E. Torres, Daniela E. Armas, Rodolfo Zanella

**Affiliations:** Instituto de Ciencias Aplicadas y Tecnología, Universidad Nacional Autónoma de México, Circuito Exterior S/N, C. U., 04510 Mexico City, México

**Keywords:** Ruthenium, TiO_2_, C_3_H_8_ oxidation, C–H bond dissociation

## Abstract

Ruthenium is a robust catalyst for a variety of applications in environmental heterogeneous catalysis. The catalytic performance of Ru/TiO_2_ materials, synthesized by using the deposition precipitation with urea method, was assessed in the catalytic oxidation of C_3_H_8_, varying the ruthenium loading. The highest catalytic reactivity was obtained for a Ru loading of 2 wt. % in comparison with the 1, 1.5, 3, and 4 wt. % Ru catalysts. The physicochemical properties of the synthesized materials were investigated by XRD, N_2_ adsorption, TEM, FT-IR pyridine, H_2_-TPR, and XPS. The size of ruthenium particles was found to be greatly dependent on the pretreatment gas (air or hydrogen) and the catalytic activity was enhanced by the small-size ruthenium metal nanoparticles, leading to changes in the reduction degree of ruthenium, which also increased the Brönsted and Lewis acidity. Metal to support charge transfer enhanced the reactant adsorption sites while oxygen vacancies on the interface enabled the dissociation of O_2_ molecules as revealed through DFT calculations. The outstanding catalytic activity of the 2Ru/TiO_2_ catalysts allowed to convert C_3_H_8_ into CO_2_ at reaction temperatures of about 100 °C. This high activity may be attributed to the metal/support interaction between Ru and TiO_2_, which promoted the reducibility of Ti^4+^/Ti^3+^ and Ru^4+^/Ru^0^ species, and to the fast migration of TiO_2_ lattice oxygen in the catalyst. Furthermore, the Ru/TiO_2_ catalyst exhibited high stability and reusability for 30 h under reaction conditions, using a GHSV of 45,000 h^−1^. The underlying alkane-metal interactions were explored theoretically in order to explain the C–H bond activation in propane by the catalyst.

## Introduction

Volatile organic compounds (VOCs) are issued by gas fields, gasoline and diesel automobile exhaust gases, manufacturing plants, household products, and liquid waste. VOCs consist, among other compounds, of light alkanes that are released into the atmosphere (Ojala et al. 2011; Zheng et al. 2014; Hui et al. 2018). Hence, the removal of light alkanes is quite defiant because these molecules are highly stable and often suffer partial destruction, yielding undesired by-products that sometimes are more hazardous than the parental compounds rather than more innocuous decomposition products such as carbon dioxide and water (Zhang et al. 2016a, b; Tomatis et al. 2016; Hu et al. 2018).

The catalytic oxidation of VOCs has attracted attention because it can control their emission at moderate operating temperature with low NO_*x*_ emission (Kamal et al. 2016; Feng et al. 2018). Normally, by this means, these volatile molecules can be fully oxidized on a catalyst at temperatures much lower than those required in thermal oxidation, revealing meaningful advantages related to energy utilization and environmental benefits (Hu et al. 2018). To this end, different catalysts have been developed, including noble metal, non-noble metal oxide, and perovskite catalysts, which have been examined in the improved removal of VOCs by the catalytic oxidation route (Zhang and He 2007; Zhang et al. 2016a, b; Tang et al. 2015). Ru catalysts have been used in several oxidation processes, such as alcohol/amine oxidation, CO oxidation, and long-chain VOC degradation (Debecker et al. 2014; Hu et al. 2018). TiO_2_ catalysts supporting ruthenium oxide, synthesized through a colloidal method, were reported as active catalysts in the catalytic oxidation of C_3_H_8_ at temperatures above 200 °C (*T*_50_ = 250 °C) (Debecker et al. 2014). However, ruthenium oxide has not been extensively investigated in catalysis, despite its interesting chemistry and lower cost than platinum- and palladium-based catalysts (Yang et al. 2016). Also, it has been shown that as a catalyst, metallic ruthenium is very active in the hydrogenation of CO_2_ (Louis Anandaraj et al. 2023), in the oxidation of CO and methanol (Calzada et al. 2020, 2017), and in ammonia synthesis (Lin et al. 2019). Earlier studies have reported that ruthenium supported on rutile TiO_2_ produced highly dispersed Ru nanoparticles that are resistant to agglomeration due primarily to the lattice matching between RuO_2_ and rutile TiO_2_ (Yang et al. 2022). Strong metal-support interactions (SMSIs) are also relevant in supported ruthenium catalysts when a reducible oxide support like TiO_2_ is employed (Zhou et al. 2022). In addition, noble metal nanoparticles present a behavior pattern that is different from that of the bulk state due to a high-surface-area-to-volume ratio and quantum size effects (Diaz et al. 2022). A peculiar relationship between the particle size and catalytic activity has been observed in Au, Pd, and Pt and catalysts (Subhan et al. 2022). Therefore, as informed in an earlier study for fcc and hcp Ru nanoparticles, the particle size exerted an effect on the catalytic activity for CO oxidation (Kusada et al. 2013). Nevertheless, the reports about the performance of Ru catalysts in the oxidation of light alkanes are very scarce (Hu et al. 2018; Debecker et al. 2014). It has been claimed that propane oxidation can proceed through isopropyl oxidation over Ru nanoparticles supported on ceria or that terminal methyl groups can be oxidized at the Ru–CeO_2_ interface, forming acrylate-type intermediates that evolve to CO_2_ and H_2_O as full oxidation products (Hu et al. 2018). However, as far as it is known, further theoretical studies have not yet been reported on the adsorption modes of light alkanes over Ru/TiO_2_ or on the study of the catalytic sites in order to rationalize the catalytic activity or reaction mechanism.

Although there are different studies on supported Ru-based catalysts, there are few research works focused on the effect exerted by the active phase-support interactions of Ru–Ti oxide-based catalysts (Zhou et al. 2022) on the catalytic oxidation of C_3_H_8_. On this basis, the aim of this work was to investigate the behavior of Ru/TiO_2_ as a model catalyst in the catalytic oxidation of C_3_H_8_. The catalytic performance features controlling the oxidation of C_3_H_8_ in the Ru/TiO_2_ catalysts were mainly addressed experimentally from aspects such as the presence of ruthenium species, ruthenium dispersion, and surface Brönsted-Lewis acidity present in the Ru/TiO_2_ catalysts in conjunction with ab initio calculations. The adsorption of propane by Ru nanoparticles supported on TiO_2_ and the activation of O_2_ were explored by DFT calculations in order to gain an atomistic understanding on the catalytic sites and electronic properties that might explain the catalytic activity.

## Experimental section

### Preparation of Ru/TiO_2_ catalysts

Ruthenium deposition was carried out by means of the deposition precipitation with urea method (DPU) to achieve nominal loadings of 1, 1.5, 2, 3, and 4 wt. % on TiO_2_, previously prepared by the sol–gel method and treated at 500 °C under air atmosphere. The ruthenium precursor RuCl_3_–3H_2_O (4.2 × 10^−3^ M) and urea (0.42 M) were dissolved in distilled water. Thereupon, 0.5 g of support was aggregated to this solution under constant stirring. After that, the temperature was increased to 80 °C and remained constant for 16 h; the reaction was performed in a double-walled batch reactor with recirculation. Following the deposition precipitation with urea method procedure, the materials were washed four times with distilled water at 25 °C, centrifuged, and vacuum dried for 2.5 h at 80 °C. The materials were named as XRu/TiO_2_, where X represents the Ru loading.

### Catalytic activity measurements

The catalytic tests were carried out in a tubular reactor with porous plate (ID = 1 cm; *L* = 35 cm) and prior to the catalytic tests, the samples underwent an activation process at 300 and 500 °C under H_2_ flow (1 mg = 1 mL min^−1^). Flow mass controllers were used to set the flow of a gas mixture at 5000 ppm of C_3_H_8_ (purity of 99.998%) and 2% of O_2_ in N_2_ (purity of 99.997%) with a total flow rate of 100 mL min^−1^. The catalyzed reaction was performed using 40 or 80 mg of Ru/TiO_2_ catalyst, from room temperature to 500 °C, with a heating ramp of 3 °C min^−1^. The effluents were analyzed by gas chromatography in an in-situ Research Rig-150 equipment; the effluent analysis was performed using an Agilent Technologies 6890N chromatograph with CO, CO_2_, and C_3_H_8_ detection. Propane conversion (%) = ([C_3_H_8_]_in_ − [C_3_H_8_]_out_*/*[C_3_H_8_]_in_) × 100.

### Characterization of catalysts

X-ray diffraction (XRD) patterns of the materials were carried out on a Bruker Advance D8 diffractometer and a graphite secondary-beam monochromator. Data were collected for dispersion angles (2*θ*) ranging from 4 to 80° with a pitch size of 0.02° for 2 s/step.

The transmission electron microscopy (HRTEM) analyses of the materials were recorded with a JEOL 2200FS microscope operating at 200 kV.

The surface areas of the materials were measured on a Micromeritics ASAP-2000 analyzer by N_2_ adsorption at − 196 °C and calculated by the BET method.

Hydrogen temperature–programed reduction of the materials was obtained in an automated catalyst characterization system AutoChem II 2920 equipped with a thermal conductivity detector under a gaseous mixture flow of 10% H_2_/Ar (25 mL min^−1^) with a heating rate of 10 °C min^−1^ from room temperature to 600 °C.

The pyridine thermo-desorption was obtained in a Nicolet 8700 spectrophotometer with a resolution of 4 cm^−1^, accumulating 50 scans. In the cell, all the samples were treated under vacuum at 400 °C for 1 h. Assessments were conducted from room temperature to 400 °C. The acidity per unit area for the Lewis and Brönsted sites was calculated following a previously reported procedure (Zholobenko et al. 2020).

X-ray photoelectron spectroscopy (XPS) spectra were obtained on a Thermo VG Scientific Escalab 250 spectrometer equipped with a hemispheric electron analyzer and an Al Kα (1486.6 eV) radiation source powered at 20 kV and 30 mA. The binding energy (BE) was determined using carbon C (1 s) as the reference line (284.6 eV).

### Computational details

Electronic and adsorption properties of ruthenium clusters adsorbed over a TiO_2_ anatase surface were studied through the spin-polarized density functional theory (DFT). The projector augmented-wave (PAW) method (Blöchl 1994; Kresse and Joubert 1999) with a plane wave basis set (with a kinetic-energy cutoff of = 600 eV) was used. DFT + U calculations were performed by using the Perdew-Burke-Ernzerhof for solids (PBEsol) functional in conjunction with the Hubbard (*U*) correction in the Dudarev formalism (Dudarev et al. 1998). A value of *U* = 3 was applied to the 3d states of titanium (Torres et al. 2021) as previously reported. The D3 (BJ) Grimme dispersion correction was included (Grimme et al. 2011). Γ-centered calculations were performed with a Gaussian smearing (*σ*) value of 0.05 eV. The calculations were converged to 10^−6^ eV in the total energy and 0.01 eV/Å on atomic forces. This method has proven to be effective for the description of TiO_2_ anatase (Torres et al. 2021). DFT calculations were carried out using Vienna Ab-initio Simulation Package VASP code (version 5.4.4) (Kresse and Hafner 1993; Kresse and Hafner 1994; Kresse and Furthmüller 1996a, b). Quantum ATK (Nanolab, v R-2020.09) (Stradi et al. 2017) and Vesta software (Momma and Izumi 2011) packages were used to display the structural models of the catalysts and for the visualization of the electronic properties. Bader charges were computed with the code developed by the Henkelman group (Henkelman et al. 2006).

A titania (001) surface was modeled through a 4 × 4 × 1 supercell with vacuum > 15 Å. The lattice parameters were fixed at the optimized values of bulk anatase as reported in Torres et al. (2021). The four bottom layers of the TiO_2_ surface were only fixed, while the positions of the remaining atoms were relaxed. A truncated octahedral ruthenium cluster with 13 atoms was optimized and adsorbed over the TiO_2_ surface.

The oxygen vacancy formation energy was computed as described in Camposeco et al. (2022a). The hydrogen adsorption energy that can be related to acid–base properties of the catalysts was calculated as described in a previous contribution and as outlined there, 17 oxygen atoms were added to build the fully oxidized metal/TiO_2-*x*_ interface (Camposeco et al. 2022b).

## Results and discussion

### Catalytic performance of Ru/TiO_2_ catalysts in the C_3_H_8_ oxidation

**Fig. 1:**
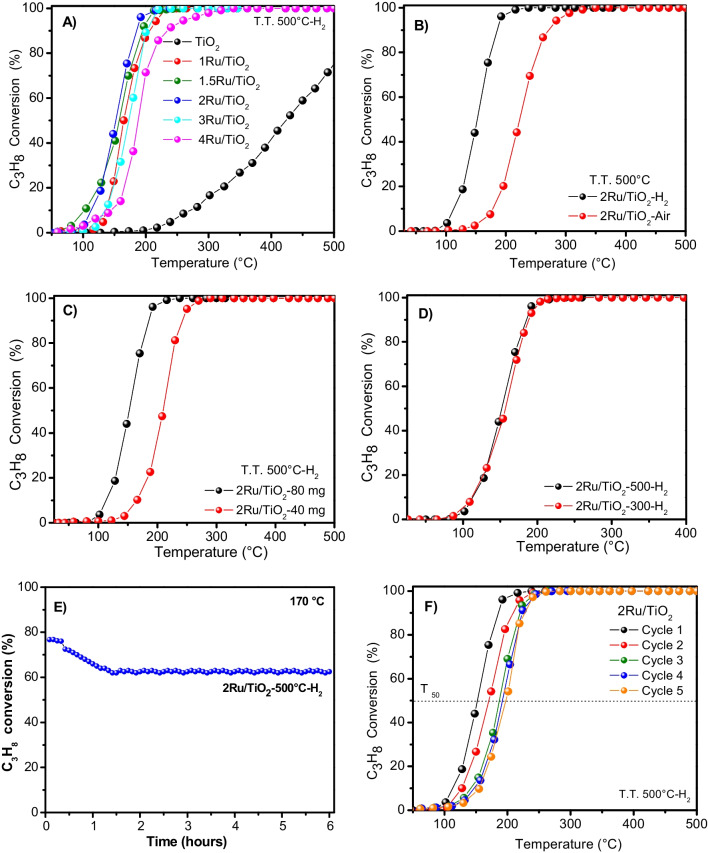
**A** C_3_H_8_ oxidation activities for the Ru/TiO_2_ catalysts with GHSV of 45,000 h^−1^ and pretreated under H_2_ atmosphere at 500 °C; **B** C_3_H_8_ oxidation conversion for the 2Ru/TiO_2_ catalyst pretreated in air or hydrogen atmosphere at 500 °C; **C** C_3_H_8_ conversions for** 2**Ru/TiO_2_ at different catalyst amounts and treated under air atmosphere at 500 °C; **D** C_3_H_8_ conversion for the 2Ru/TiO_2_ catalyst treated at 300 and 500 °C under H_2_ atmosphere; **E** deactivation test for the 2Ru/TiO_2_ catalyst under H_2_ atmosphere at 500 °C under dry conditions; and **F** reusability test for the Ru/TiO_2_ catalyst under H_2_ atmosphere at 500 °C under dry conditions

Figure 1A shows the light off curves for the total C_3_H_8_ oxidation. The 2Ru/TiO_2_ catalyst started to be active at 100 °C, reaching 90% of propane conversion at a temperature (*T*_90_) of 185 °C, while the 1Ru/TiO_2_, 1.5Ru/TiO_2_, 3Ru/TiO_2_, and 4Ru/TiO_2_ catalysts displayed *T*_90_ values of 190, 192 °C, 195 and 205 °C, respectively. The temperatures to reach 50% of propane conversion (*T*_50_) were 164, 157, 150, 173, and 190 °C for the 1Ru/TiO_2_, 1.5Ru/TiO_2_, 2Ru/TiO_2_, 3Ru/TiO_2_, and 4Ru/TiO_2_ catalysts, severally. The highest conversion was obtained for the Ru loading of 2 wt. %; higher or lower Ru loadings displaced the propane oxidation to higher temperatures, being the decrease more evident for the higher ruthenium loadings. As expected, bare TiO_2_ exhibited quite poor activity in the catalytic oxidation of C_3_H_8_, achieving 50% of conversion at about 400 °C. By using Ru/TiO_2_ catalysts, only carbon dioxide and water were obtained without any other by-products, corroborating that the ruthenium catalysts are totally selective to carbon dioxide, and that the catalytic activity was closely related to the ruthenium loading.

The TOF, reaction rate, and apparent activation energy (*E*_a_) of the Ru/TiO_2_ catalysts were estimated, and the outcomes are shown in Table 1, where it is observed that the TOF and reaction rates of the 2Ru/TiO_2_ catalysts exceeded the ones of the 1Ru/TiO_2_, 1.5Ru/TiO_2_, 3Ru/TiO_2_, and 4Ru/TiO_2_ catalysts. In the same way, the lowest *E*_a_ value was obtained by the sample containing 2 wt. % of Ru (as shown in Table 1).

**Table 1:** Structural and catalytic properties of the Ru/TiO_2_ catalysts thermally treated at 500 °C under hydrogen atmosphere

Sample	Metal Size (nm) by TEM	Crystallite size (nm)	Rate _C3H8_ mol (g_metal_·s)^−1^_110 °C_	*E*_a C3H8_ (kJ mol^−1^)	Dispersion (%)	TOF (s^−1^) _at 110 °C_
1Ru/TiO_2_	2.2	15	2.4 * 10^−4^	90	46	5.2 * 10^−2^
1.5Ru/TiO_2_	2.0	16	3.1 * 10^−4^	84	48	5.7 * 10^−2^
2Ru/TiO_2_	1.6	16	3.3 * 10^−4^	79	58	6.5 * 10^−2^
3Ru/TiO_2_	2.8	18	3.0 * 10^−4^	82	35	5.4 * 10^−2^
4Ru/TiO_2_	3.8	19	2.1 * 10^−4^	94	30	4.1 * 10^−2^

To study the effect of the pretreatment gas, air or hydrogen was employed during the thermal treatment of the 2Ru/TiO_2_ catalyst. In Fig. 1B, it is shown that the gas atmosphere used for the thermal treatment has a great influence on the catalytic oxidation of propane. The catalytic activity of the 2Ru/TiO_2_ catalyst heated in the presence of a flow of H_2_ is much higher than that of the catalyst heated in a flow of air, showing a difference of 100 °C in the *T*_50_. It is shown in the following sections that when hydrogen is used, ruthenium in the 2Ru/TiO_2_ catalyst is mainly reduced to Ru^0^, while when that catalyst was treated in air, the presence of Ru^2+^ species was identified (see XPS results below), suggesting that Ru is more active in the metallic than in the oxidized state for propane oxidation. As the catalytic oxidation of propane was remarkably different as a function of the gas used for the thermal treatment, it can be proposed that the particle dispersion and oxidation state of ruthenium exert an important effect on the propane conversion and as consequence on the catalytic activity, in agreement with Orendorz et al. (2005). Moreover, the outstanding interaction between metallic ruthenium and TiO_2_, when the sample was thermally treated in H_2_, may improve the catalytic oxidation of propane according to an earlier work (Chen et al. 2012).

The gas hourly space velocity (GHSV) effect was evaluated for the 2Ru/TiO_2_ catalyst, using 40 or 80 mg corresponding to 86,000 and 45,000 h^−1^ as shown in Fig. 1(C). As expected, the C_3_H_8_ conversion of the 2Ru/TiO_2_ catalyst was superior for a GHSV of 45,000 h^−1^ because the residence time was higher.

The effect of the annealing temperature for the 2Ru/TiO_2_ catalyst was evaluated at 300 and 500 °C. Figure 1D shows that *T*_90_ was reached at 220 and 200 °C for thermal treatment at 300 and 500 °C, respectively. It is observed that the C_3_H_8_ conversion is slightly higher after a pretreatment at 500 °C, which is explained because at temperatures above 300 °C, ruthenium should be found mainly as a combination of Ru^0^/Ru^2+^ species. The fact that the C_3_H_8_ oxidation rate was slightly enhanced with the pretreatment at 500 °C in H_2_ agrees with the proposal that metallic species of ruthenium are the active sites in oxidation reactions (Hu et al. 2018).

On the other hand, the stability test for the 2Ru/TiO_2_ catalyst pretreated at 500 °C in H_2_ was analyzed at a reaction temperature of 170 °C (Fig. 1E). The C_3_H_8_ conversion under reaction conditions for 5 h was observed to decrease from 78 to 60%; this slight deactivation may be due to the rearrangement of ruthenium particles on the surface of the TiO_2_ catalysts under the initial conditions. Also, the TiO_2_ anatase structure could be affected by the reaction temperature due to carbon deposition in agreement with Camposeco et al. (2022a, b). The reusability test of the 2Ru/TiO_2_ catalyst, thermally treated at 500 °C under hydrogen atmosphere, was assessed through five consecutive reaction cycles, as depicted in Fig. 1F. The results revealed a slight shift toward higher reaction temperatures over the course of the five reaction cycles. *T*_50_ conversion at 150 °C was reached in the first run. However, in the subsequent runs (i.e., second, third, fourth, and fifth), the *T*_50_ conversions were at 169, 184, 190, and 195 °C, respectively. Furthermore, it is important to highlight that after completing the five reaction cycles, complete transformation of C_3_H_8_ into CO_2_ was achieved at 270 °C. These variations in the reaction temperature can be attributed to the potential rearrangement of Ru nanoparticles on the TiO_2_ surface or changes in the oxidation state of ruthenium under the reaction conditions, as revealed by the characterization of the spent catalysts in the corresponding section below.

### Structural and textural properties of Ru/TiO_2_ catalysts

**Fig. 2:**
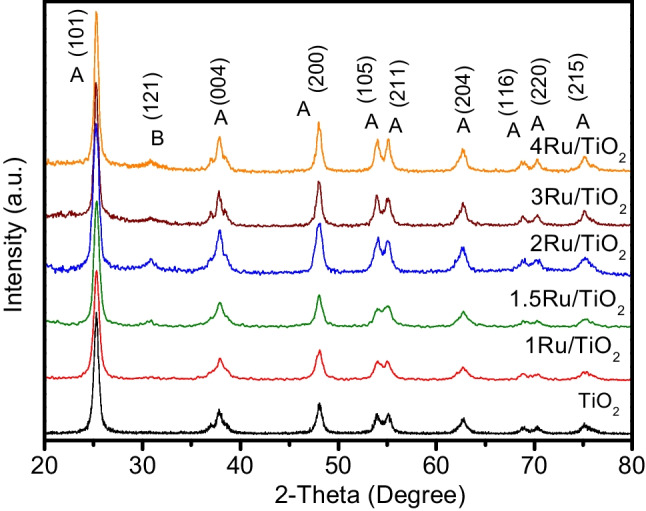
XRD patterns for the Ru/TiO_2_ sample thermally treated at 500 °C under hydrogen atmosphere

A representative series of XRD patterns for the Ru/TiO_2_ catalysts is displayed in Fig. 2. After the addition of ruthenium by the deposition precipitation with urea method, no peaks corresponding to Ru were detected on TiO_2_ according to the JCPDS card: 06–0663. The outcomes suggested a high dispersion of ruthenium on the TiO_2_ supports Hu et al. (2018) and Debecker et al. (2014). With regard to TiO_2_, the observed diffraction peaks were assigned to orthorhombic brookite TiO_2_ (JCPDS card: 29–1360). It is worth noting that as the ruthenium loading increased, a slight growth in the brookite phase at 2*θ* = 30.8° (121) was observed for all the samples containing ruthenium. Moreover, the peaks at 2*θ* = 25.37° (101), 37.80° (004), 48.04° (200), 53.89° (105), 62.68° (204), 68.76° (116), 70.30° (220), and 75.02° (215) were mainly assigned to the tetragonal anatase TiO_2_ phase (JCPDS card: 21–1272). Additionally, the TiO_2_ crystallite size was calculated by the Scherrer equation at 2*θ* = 25.37° (101), revealing crystallite sizes between 15 and 19 nm, which confirmed that the presence of ruthenium on TiO_2_ did not affect evidently the textural and structural properties of TiO_2_, see Fig. 2.

**Fig. 3:**
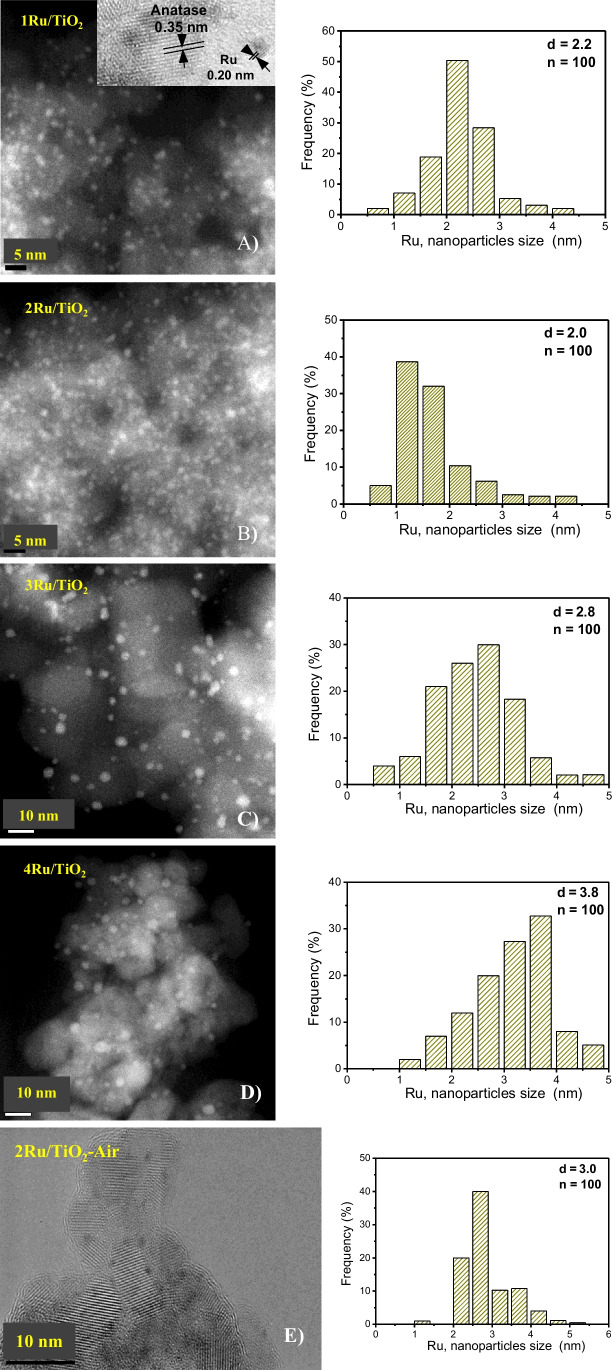
Microscopy images for the catalysts thermally treated at 500 °C under H_2_ atmosphere: **A 1**Ru/TiO_2_ catalyst and its corresponding frequency histogram, **B 2**Ru/TiO_2_ catalyst and frequency histogram, **C 3**Ru/TiO_2_ catalyst and respective frequency histogram, **D 4**Ru/TiO_2_ catalyst and its corresponding histogram, and **E 2**Ru/TiO_2_ catalyst calcined at 500 °C under air atmosphere and frequency histogram

TEM and HRTEM images of the Ru/TiO_2_ catalysts are featured in Fig. 3. The particles with a lattice fringe spacing of 0.206 nm are related to ruthenium (101) (JCPDS: 06–0663), see inset in Fig. 3A. Hence, the lattice fringe spacing of 0.352 nm for the 1Ru/TiO_2_ catalyst is assigned to anatase (101) (JCPDS: 21–1272). This indicates that metallic ruthenium was successfully loaded and dispersed on the titanium dioxide surface using the deposition precipitation with urea method. In Fig. 3(A–D), the corresponding particle size histograms are also displayed for the 1Ru/TiO_2_, 2Ru/TiO_2_, 3Ru/TiO_2_, and 4Ru/TiO_2_ samples thermally treated in hydrogen. The 1Ru/TiO_2_ catalyst shows ruthenium particle sizes of ~ 2.2 nm, while for 2Ru/TiO_2_, the particle size is ~ 1.6 nm; for 3Ru/TiO_2_, the particle size is ~ 2.8 nm; and for 4Ru/TiO_2_, the particle size is ~ 3.8 nm. These sizes are according to XRD outcomes, where no ruthenium diffraction peaks were detected, because during the synthesis of ruthenium, highly dispersed particles were formed (Hu et al. 2018; Debecker et al. 2014). These results suggest that the deposition precipitation with urea method is suitable for depositing very small ruthenium particles on the TiO_2_ surface even when up to 4 wt. % of Ru was used. Figure 3(E) displays a TEM image and the ruthenium size histogram of the 2Ru/TiO_2_ material treated under air atmosphere. It is observed that larger ruthenium particles of 3.0 nm were formed on the TiO_2_ surface, which differs in the catalysts thermally treated under hydrogen. The Ru loading on TiO_2_ induced slight modifications in the metal particle size (Table 1). At loadings between 1 and 2 wt. %, the Ru particle size remained practically constant around 2 nm. However, loadings above 2 wt. % resulted in a slight increase in Ru particle size. This could be attributed to enhanced interaction of metal precursors during the deposition–precipitation with urea method on TiO_2_ at higher loadings, leading to increased Ru agglomeration during thermal treatment.

### Reducibility, acidity, and oxidation state of Ru/TiO_2_ catalysts

**Fig. 4:**
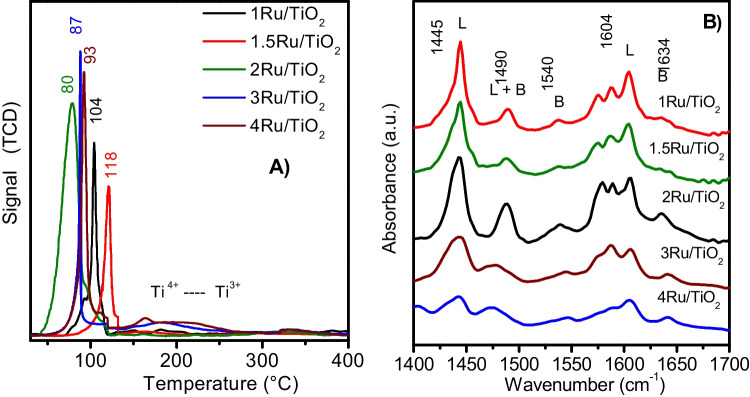
**A** Reduction profiles of the Ru/TiO_2_ catalysts and **B** FTIR thermo-desorption of pyridine of the Ru/TiO_2_ catalysts at 200 °C

The reduction evolution of a series of Ru/TiO_2_ catalysts with distinct ruthenium amounts was studied by H_2_/TPR experiments as shown in Fig. 4, where two reduction peaks are mainly observed, which are related to the reduction of diverse chemical species of ruthenium on titanium dioxide. The first peak could be associated with the reduction of RuO_2_ oxide, whereas the second one has been correlated to the reduction of RuO_2_ to the Ru^0^ interphase on TiO_2_ (Hernandez-Mejia et al. 2016). Outcomes from the H_2_ consumption for the samples with Ru loadings of 1, 1.5, 2, 3, and 4 wt. % show an increase in H_2_ consumption as the ruthenium charge grew, with values of 2.8, 3.1, 3.6, 2.2, and 1.9 times higher than the stoichiometric estimated values, respectively; these results indicate the co-reduction of the RuO_2_/TiO_2_ interphase, which agrees with earlier results (Taylor 1975; Maumela et al. 2021). In effect, well-dispersed ruthenium on TiO_2_ tends to generate stronger interaction. Likewise, the increase in metal loading provoked a shift to a lower reduction temperature of RuO_2_ (Fig. 4A).

The FTIR spectra of thermo-desorption of pyridine adsorbed on the Ru/TiO_2_ catalysts with distinct ruthenium amounts are shown in Fig. 4B. According to the literature, typical bands located at 1445, 1490, and 1540 cm^−1^ are ascribed to pyridine adsorbed on Lewis acid sites, on both Lewis and Brönsted, and on Brönsted acid sites, severally (González et al. 2017; Bordiga et al. 2015). A typical band located at 1575 cm^−1^ is related to physically adsorbed pyridine, while hydrogen-bound pyridine with surface hydroxyl groups is revealed by the characteristic bands located at 1600 and 1635 cm^−1^. Thereby, previous studies on surface acidity of the TiO_2_ anatase phase only showed Lewis acid sites, while bands stemming from Brönsted acid sites were not observed after 100 °C (Phung et al. 2015; Busca 2019). Mostly, the acid sites on the surface of the catalyst can promote the adsorption of C_3_H_8_ and C–H bond breaking during the reaction (Zhao et al. 2020). These results suggest that the incorporation of ruthenium to TiO_2_ results in a considerable increase in Lewis acidity and in the occurrence of Brönsted acid sites, while raising the ruthenium amount to 2 wt. % caused a small enhancement in the Brönsted acidity, see Table 2. So, both Lewis and Brönsted sites co-exist on the Ru/TiO_2_ catalysts and consequently, they have an important participation in the total oxidation of propane, in agreement to previous works (Dimitratos and Védrine 2006; Zhang et al. 2021).

**Table 2:** Surface area and surface Brönsted and Lewis acidity of the Ru/TiO_2_ catalysts

Sample	S_BET_ (m^2^ g^−1^)	*C*_Lewis_ (µ-mol m^−2^)	*C*_Brönsted_ (µ-mol m^−2^)	*C*_Total_ (µ-mol m^−2^)	Desorption temperature (°C)
TiO_2_	88	0.90 0.65	0.11 0.05	1.01 0.70	100 200
1Ru/TiO_2_	85	1.20 0.90	0.11 0.08	1.31 0.98	100 200
1.5Ru/TiO_2_	80	1.30 0.95	0.20 0.10	1.50 1.05	100 200
2Ru/TiO_2_	78	2.20 1.20	0.38 0.17	2.58 1.37	100 200
3Ru/TiO_2_	70	1.50 0.95	0.16 0.09	1.66 1.04	100 200
4Ru/TiO_2_	62	1.30 0.91	0.15 0.08	1.45 0.99	100 200

**Fig. 5:**
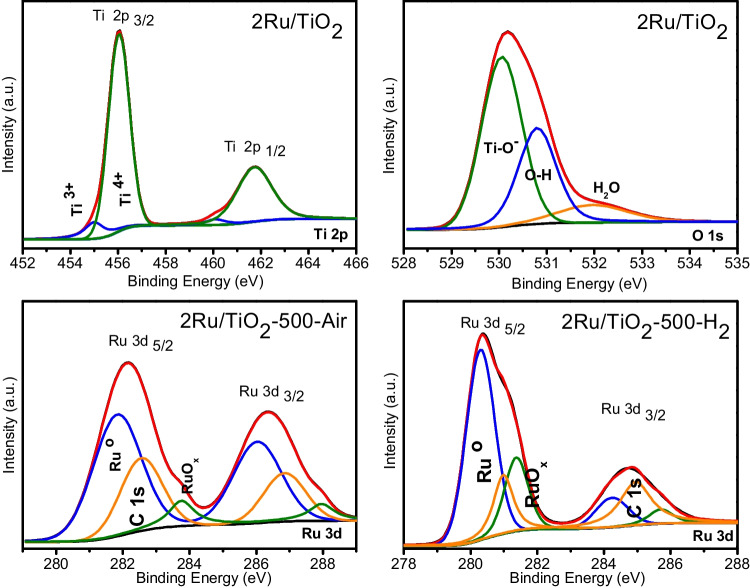
X-ray photoelectron spectroscopy spectra for the titanium Ti 2p, oxygen O 1 s, and ruthenium Ru 3d for the 2Ru/TiO_2_ catalyst thermally treated at 500 °C

The XPS outcomes obtained from the sample containing 2 wt. % of ruthenium on the TiO_2_ support are presented in Fig. 5. In this sense, the chemical valence of the ruthenium species seems to be a determining factor in the C_3_H_8_ catalytic activity of these materials. Therefore, ruthenium 3d spectra show two different species: Ru^0^ at 281.3 eV and Ru^4+^ at 282.8 eV. The presence of Ru^4+^ species indicates that ruthenium is found as RuO_2_. As mentioned in an earlier work (Bock et al. 2004), the Ru3d_5/2_ (≈ 281 eV) signal overlaps with that of C1s (≈ 284 eV), which makes difficult to resolve the small Ru 3d_5/2_ peak when compared to the one of C1s (Moulder et al. 1992). The Ru^0^ and Ru^4+^ species were predominant on the surface of the Ru/TiO_2_ catalysts, which was consistent with the results reported in the literature (Bock et al. 2004). These results showed that the flow gas used during the thermal treatment (air or H_2_) significantly affected the species present in the Ru/TiO_2_ catalysts, as depicted in Figs. 1B and 5. Oxygen 1 s spectra of the 2 wt. % of ruthenium on TiO_2_ catalysts are displayed in Fig. 5. Three peaks are observed: one (O_I_) at 530.0 eV, a second one (O_II_) at 531.5 eV, and a third one (O_III_) at 532.2 eV, which are associated with lattice oxygen (Ti–O^−^), surface oxygen from adsorbed water or hydroxyl species (Ti–OH), and H_2_O, respectively (Stradi et al. 2017). In the case of titanium 2p, a wide shoulder is observed at the low binding energy (BE) side of the Ti2p_1/2_ and Ti 2p_3/2_ spectra. The deconvolution performed to the Ti 2p_3/2_ profiles revealed two subjacent components at BE of 456.5 eV and 454.9 eV, associated with titanium in its Ti^4+^ and Ti^3+^ oxidation states, respectively (Dupin et al. 2000).

### Characterization of the spent Ru/TiO_2_ catalysts

**Fig. 6:**
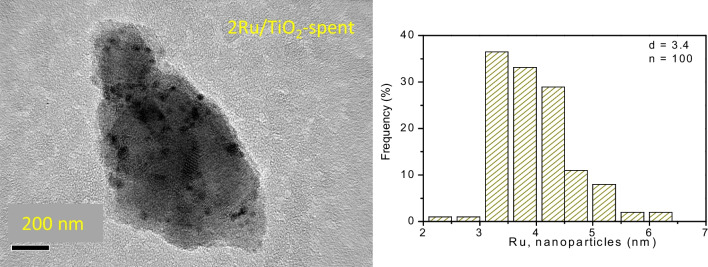
TEM image of the 2Ru/TiO_2_ catalyst thermally treated at 500 °C under hydrogen atmosphere, after C_3_H_8_ oxidation reaction and its corresponding frequency histogram

**Fig. 7:**
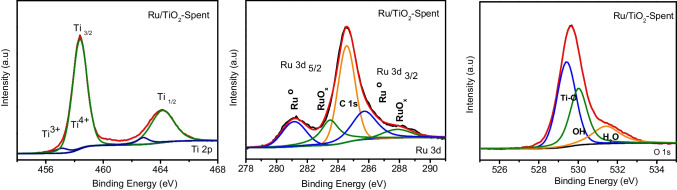
X-ray photoelectron spectroscopy spectra of the titanium Ti 2p, oxygen O 1 s, and ruthenium Ru 3d for the 2Ru/TiO_2_ catalyst, after C_3_H_8_ oxidation reaction

The 2Ru/TiO_2_ catalyst after the C_3_H_8_ oxidation reaction was characterized using TEM and XPS, as shown in Figs. 6 and 7. The TEM image of the 2Ru/TiO_2_ catalyst (Fig. 6) displayed an increase in the particle size of ruthenium from 1.6 to 3.2 nm. Prior to the C_3_H_8_ oxidation reaction, the 2Ru/TiO_2_ catalyst exhibited two split spin–orbit components associated with Ru 3d_5/2_ and 3d_3/2_ at 280.4 and 284.7 eV, respectively, representing Ru^0^ and RuO_x_ species, see Fig. 5. Subsequently, the same catalyst was characterized after the C_3_H_8_ oxidation reaction. Through the deconvolution of the split spin–orbit components of 3d_5/2_ and 3d_3/2_, a change in the Ru^0^/RuO_x_ ratio was observed. The predominance of Ru^0^ shifted to a mixture of oxidized and reduced species. These findings indicate that the loss of Ru^0^ metallic species and the growing of Ru particle size might be connected to the decrease in catalytic activity for the C_3_H_8_ oxidation reaction.

Ruthenium catalysts supported on Al_2_O_3_, CeO_2_ or TiO_2_ have been previously studied in the C_3_H_8_ catalytic oxidation reaction and some of the outcomes that have been reported are summarized in Table 3. The Ru/TiO_2_ catalysts synthesized in this work were active from lower reaction temperatures with respect to most of the Ru catalysts previously reported. In addition, they are active from lower temperatures unlike the Ru/TiO_2_ catalysts prepared by an aqueous colloidal method reported in Debecker et al. (2014) and Yang et al. (2016). However, the comparison listed in Table 3 must be taken carefully because the conditions for testing the catalysts were very different.

**Table 3:** Comparative chart of C_3_H_8_ catalytic oxidation materials

Catalyst	Ea [kJ]	Temp [°C]	Ru load metal (wt.%)	Ref
Ru/Al_2_O_3_	96	100–200	3.0	Okal and Zawadzki (2009)
Ru/CeO_2_	72	100–200	3.0	Okal and Zawadzki (2009)
Ru/TiO_2_	–	200–350	1.3	Debecker et al. (2014)
Ru/TiO_2_	79	100–200	2.0	This work

### Computational results

**Fig. 8:**
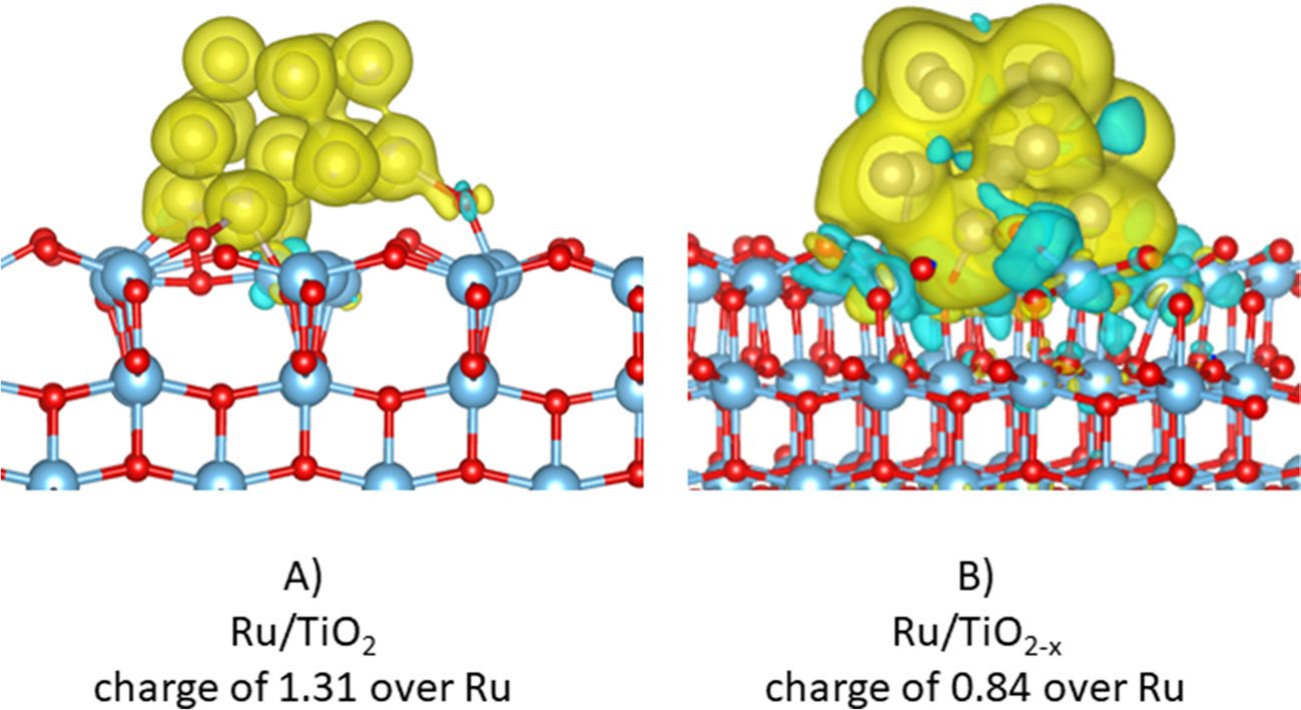
Geometries of **A** Ru/TiO_2_ structure and **B** Ru/TiO_2-x_ interface with an oxygen vacancy. Color code: Ti, white; O, red; Ru, green. The yellow lobes are charge depleted areas, while the blue ones refer to charge gain

Outcomes from DFT calculations are shown herein to analyze the acidic and electronic properties that might explain the catalytic activity of Ru nanoparticles supported on TiO_2_ in the C_3_H_8_ oxidation. A Ru_13_ cluster was adsorbed on a pristine TiO_2_-001 anatase surface (Fig. 8A) and one with a single oxygen vacancy (Fig. 8B), comparatively. The presence of the defective sites might be favored under reducing conditions (H_2_ gas atmosphere) and it slightly improved the ruthenium-titania interaction, since the computed adsorption energy is − 15.2 eV over TiO_2_, while that on TiO_2-*x*_ is − 15.6 eV. A charge of 0.84 over Ru atoms on TiO_2-*x*_ is found, therefore showing that the ruthenium cluster transferred charge to the titania surface, thus enhancing its reducibility. These results are consistent with the improved efficiency of the catalyst under H_2_ gas pretreatment.

As aforementioned, from the experiments, it was found that the Ru/TiO_2_ catalyst presents Lewis acid sites that are prone to attract electron donor species such as water molecules instead of oxygen. H_2_O was detected by XPS in the catalyst treated thermally at 500 °C; then, in order to explore if it can hinder Lewis acid catalytic sites, the comparative adsorption properties of O_2_ and H_2_O were computed theoretically.

**Fig. 9:**
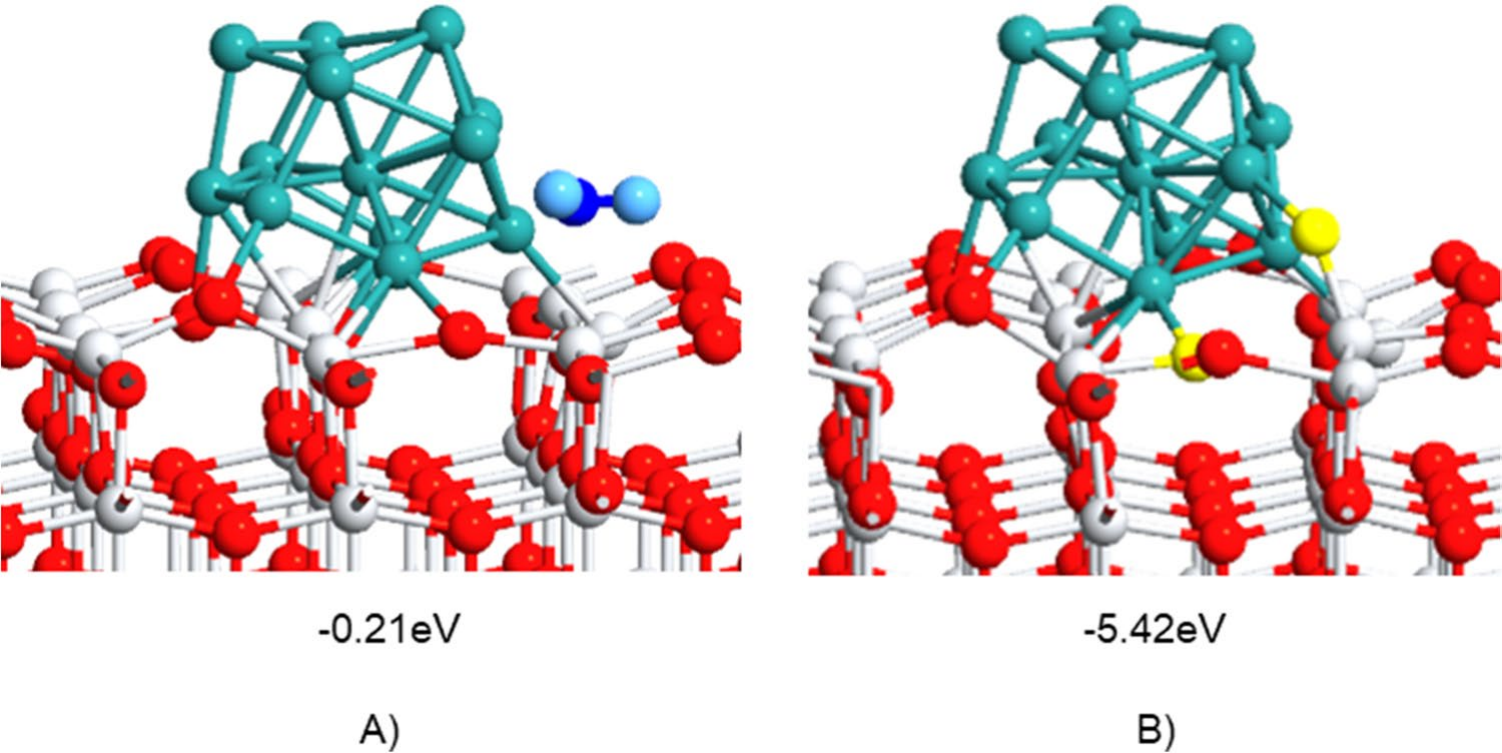
Geometries of Ru/TiO_2-2×_ structures showing adsorption modes of **A** water or **B** O_2_ molecules in the interface (Ru/TiO_2-*x*_O). The computed adsorption energies are presented. Color code: Ti, white; O, red; Ru, green; H_2_O, blue; O_2_, yellow

For that, a second oxygen vacancy was set up in the Ru/TiO_2-*x*_ interface, since this defect might act as an oxygen activation site. The oxygen atom to be pulled out was picked given its larger oxygen-neighboring atom bond lengths. Then, an oxygen molecule was adsorbed on this defective site to study its activation. The results showed that the O_2_ molecule adsorbed on the interface displayed dissociatively a resultant O–O bond distance of 2.81 Å in which one of the oxygen atoms occupied the vacant site, whereas the other was bound to Ru and Ti atoms as depicted in Fig. 9B.

Interestingly, the adsorption of O_2_ (adsorption energy =  − 5.42 eV) is preferred over water adsorption (adsorption energy =  − 0.21 eV) on the interface, which arises from the comparison between the computed adsorption energies for both molecules (Fig. 9). A water molecule bound through the oxygen atom to Ti and Ru atoms on the interface was found to have an average bond distance of 2.37 Å (see Fig. 9A). These results suggest that Ru-containing catalysts might be water tolerant in catalysis.

Moreover, the oxygen adsorption on the Ru-titania interface resulted thermodynamically highly favored when compared to analog Pt- or Pt–Pd-containing structures (Camposeco et al. 2022b). Then, higher oxygen adsorption energy (more negative) might be indicative of stronger Lewis acidity, thus being the Ru cluster the most Lewis-acidic one.

Studies of alkane activation over metal oxides have shown that the C–H bond activation proceeds through a molecular adsorbed precursor prior to the bond scission (Weaver et al. 2014; Martin et al. 2021). It is claimed that these intermediates correspond to sigma complexes that are formed through dative bonding interactions among alkane molecules and under-coordinated metal atoms (Martin et al. 2021). Then, it is relevant to address the propane-NP/TiO_2_ interaction to get insight into the initial alkane activation mechanism. Besides, it has been found that hydrogen abstraction occurs via a radical mechanism over V_2_O_5_ in which the C–H bond activation barrier is partially determined by hydrogen adsorption on the surface (Alexopoulos et al. 2012; Sprung et al. 2018). Likewise, it has been found that the presence of adsorbed late transition metals over reducible metal oxides modifies the Lewis acid properties of the catalyst, increasing the catalytic activity toward alkane oxidation (Lyu et al. 2021).

Then, to research the acidic characteristic of the catalyst at an atomistic level, the hydrogen adsorption energy (EH_ads_) on the oxidized catalyst interface was computed. In a molecular approach, this property is analogous to the proton affinity (PA) (with opposite sign) given by EH_ads_ =  − PA. The PA is a measure of the acid strength of species in the gas phase, where the lower the proton affinity, the stronger the acid. Then, the hydrogen adsorption energy may be an indicator of the acidic properties of the model catalysts that influence the catalytic activity. Based on this, a stronger acid site is characterized by lower (less negative) hydrogen adsorption energy (Camposeco et al. 2022b).

**Fig. 10:**
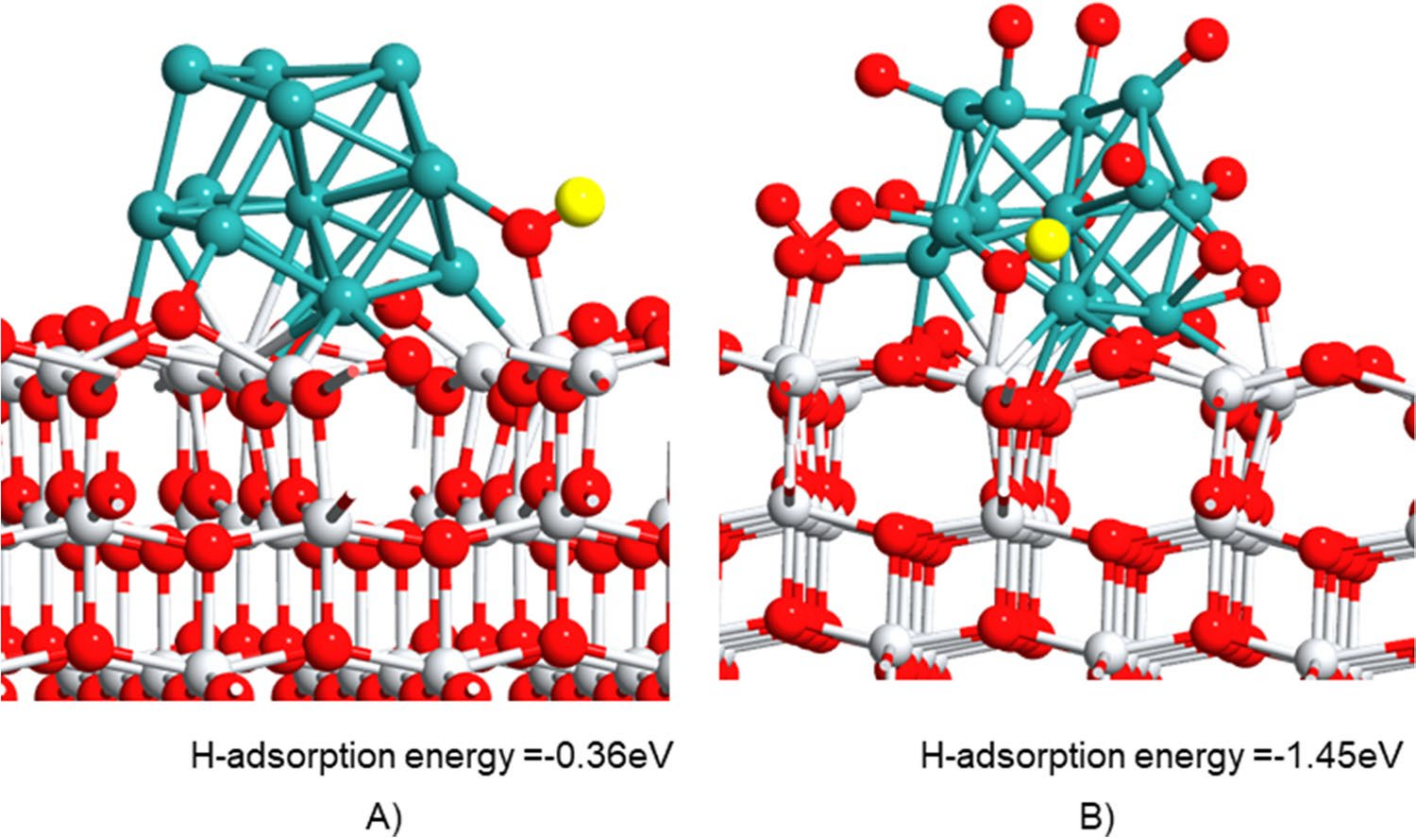
Geometries of **A** Ru/TiO_2-*x*-_O structure and **B** oxidized Ru/TiO_2-*x*-_O_16_ interface with a single hydroxylated site. Color code: Ti, white; O, red; Ru, green; H added to form OH, yellow

The added oxygen atom on the interface was hydroxylated to calculate the hydrogen adsorption energy on a single oxidized site and on an oxidized interface containing 17 oxygen atoms. The results shown in Fig. 10 revealed that the hydrogen atom preferred to be adsorbed on an oxidized interface. By following the trend of proton affinity, given the lower value of the single hydroxylated structure (Fig. 10A), it is a stronger Brönsted acid that might be more active in propane oxidation than the oxidized Ru/TiO_2-*x*-_O_16_ catalyst (Fig. 10B).

**Fig. 11:**
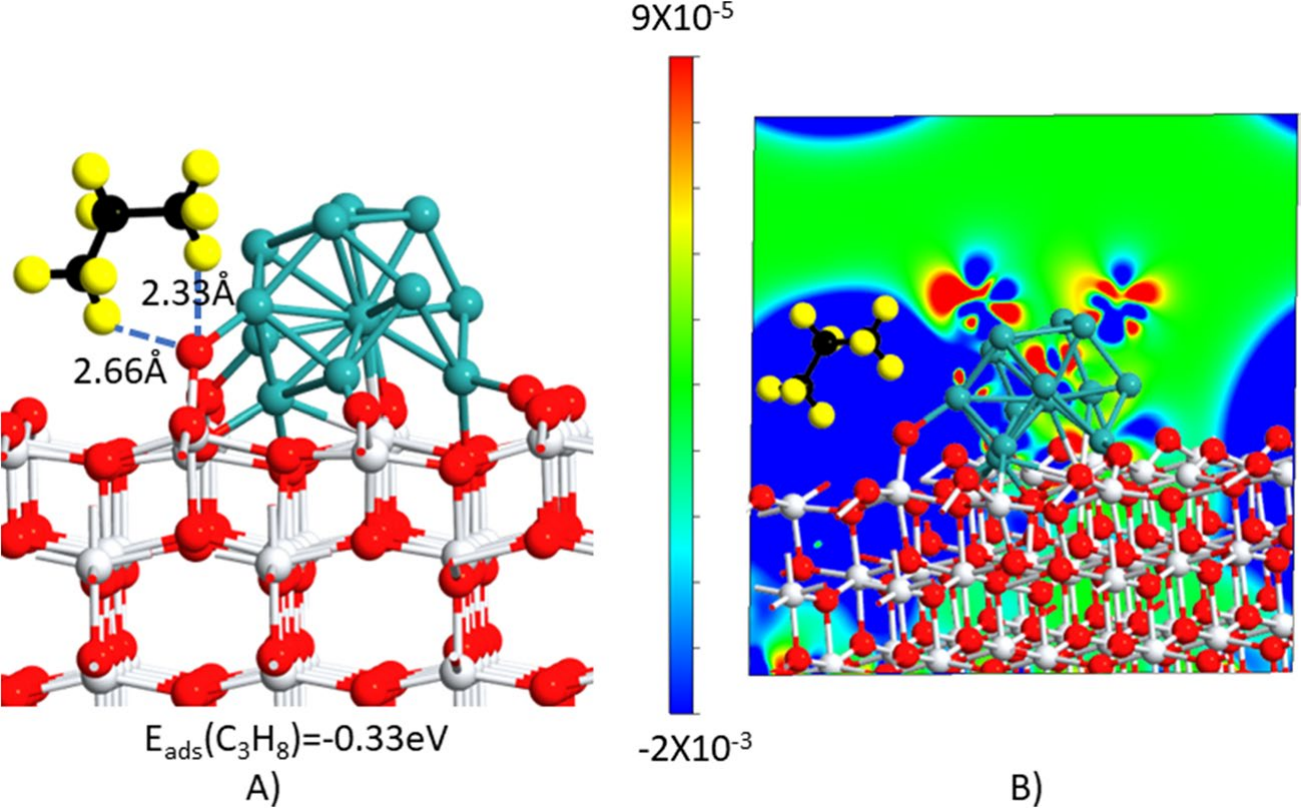
**A** Geometries of the C_3_H_8_ molecule adsorbed on Ru/TiO_2-*x*_O structures. Color code: Ti, white; O, red; Ru, green; C, black; H, yellow. Oxygen-hydrogen bond distances from the oxidized site on the interface and the adsorbed C_3_H_8_ molecule are depicted with a dotted blue line. C_3_H_8_ adsorption energies are presented in eV. **B** Corresponding charge density difference plot (electrons/bohr^3^). The blue (red) distribution corresponds to charge accumulation (depletion)

The propane adsorption on the supported NP models oxidized in a single site on the TiO_2_ surface is shown in Fig. 11. Propane interacts electrostatically with the interface as it can be seen from the computed hydrogen–oxygen distances from propane and surface oxygen (Fig. 11A), preferentially through the terminal methyl groups.

The computed physisorption energy evidenced that propane adsorption on the catalyst interface is energetically favored based on the computed adsorption energy of − 0.33 eV. This value is consistent with those reported for different metal oxides in previous studies (Martin et al. 2021; Ding et al. 2020; Chang et al. 2017; Liu et al. 2016).

**Fig. 12:**
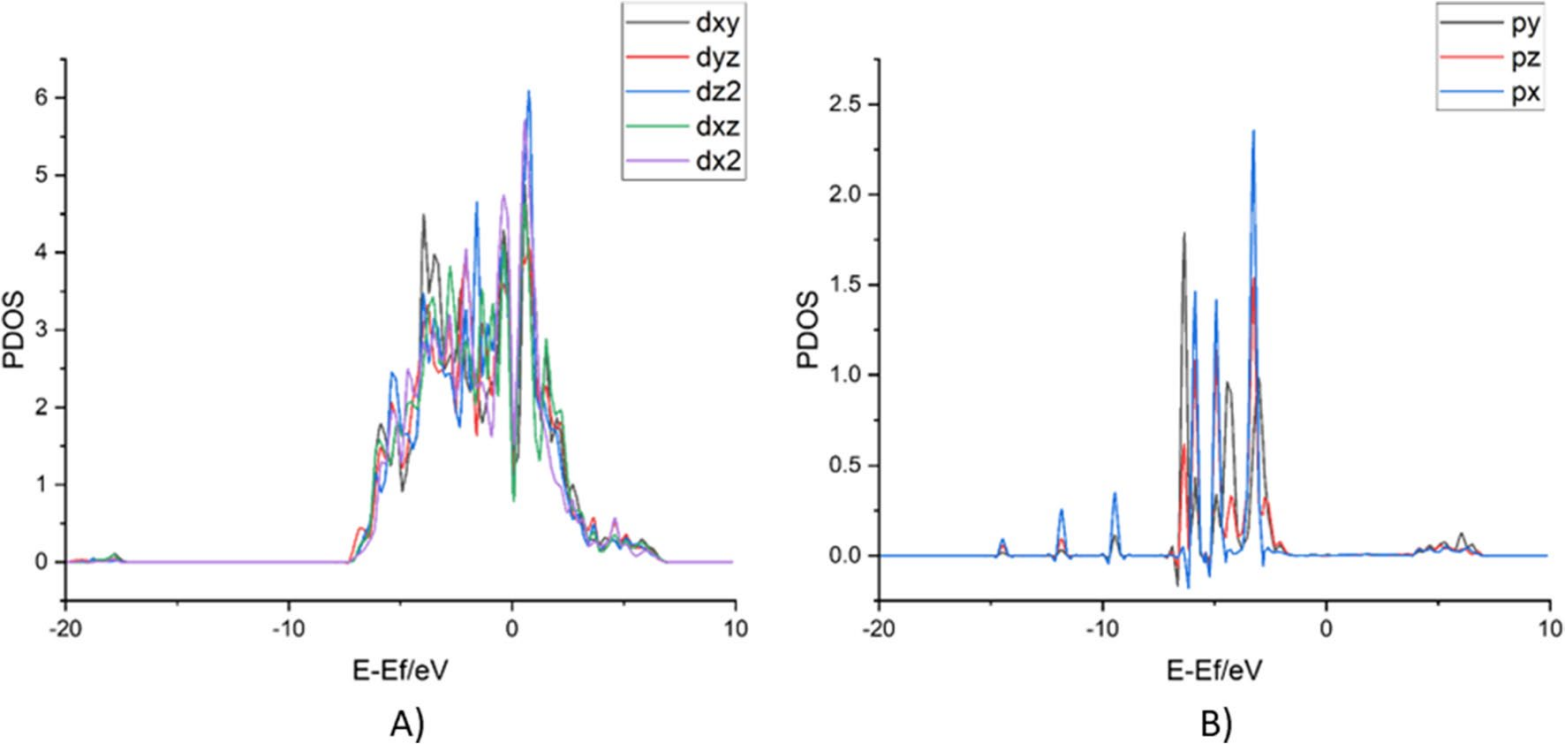
Projected density of states (PDOS) of the dominant contributions in the band edges is displayed for **A** Ru and **B** carbon from propane. The PDOS of the 4d orbitals of Ru atoms and that of the 2p orbitals of C atoms on the chemisorbed alkane as shown in Fig. 11A are displayed

The charge density difference map revealed that propane adsorption may be mediated by band interactions between C-2p filled orbitals and Ru-4d orbitals as it can be seen from the PDOS plot displayed in Fig. 12. Indeed, there are several unfilled d states near the Fermi level that might be beneficial for alkane adsorption and C–H bond activation as claimed in other studies (Martin et al. 2021; Wang et al. 2012). It has been asserted that those unoccupied states accept charge from C–H σ orbitals, thus enabling alkane interaction (Fung et al. 2020). Then, from these results, it was confirmed that Ru provided adsorption sites for propane, which through charge transfer from the cluster to the support unfilled states near the Fermi level are available for interacting with the alkane molecule, thus enabling its oxidation.

## Conclusions

Ru/TiO_2_ catalysts thermally treated in H_2_ displayed outstanding behavior in the catalytic oxidation of C_3_H_8_ at low temperature (*T* ≥ 100 °C). According to outcomes, this high catalytic performance was chiefly associated with the strong interaction between TiO_2_ and metallic Ru species. This interaction is evidenced by the charge transfer from ruthenium to reduced titania determined through ab initio calculations. Then, this increase in charge on the TiO_2_ interface enabled it to function as a reservoir for oxygen and provided extra sites for C_3_H_8_ adsorption during the reaction. Likewise, the strong titanium dioxide-ruthenium interaction produced a higher proportion of Lewis and Brönsted acid sites for a ruthenium loading of 2 wt. %; thereby, Ru/TiO_2_ enhanced the adsorption of oxygen. The theoretical results revealed that oxygen was adsorbed dissociatively on the ruthenium-titania interface and that even though water molecules can be physisorbed on the interface, it is less prone to oxidize them.

Finally, the metal–support interface promoted the C_3_H_8_ oxidation reaction. Lewis acidity of this catalyst might improve the C–H bond activation in C_3_H_8_ as determined from experimental and computational results. The alkane adsorption on the interface is promoted by interactions between d-ruthenium unoccupied states and p-carbon states from propane.

## Data Availability

Not applicable.
